# Art Is Fun, Art Is Serious Business, and Everything in between: Learning from Art Therapy Research and Practice with Children and Teens

**DOI:** 10.3390/children9091320

**Published:** 2022-08-30

**Authors:** Einat S. Metzl

**Affiliations:** Art Therapy Grad Program, Department of Jewish Art, Bar-Ilan University, Ramat Gan 5290002, Israel; einat.metzl@biu.ac.il

**Keywords:** children, adolescents, art therapy, art materials, art process, art products

## Abstract

This paper explores the current theoretical frames of working with children and adolescents, considers the socio-political and developmental considerations for art therapy practice within settings, and systems in which children are embedded. An illustration of the use of art materials, processes, and products for children and adolescents based on an art therapist’s clinical experience in school settings, mental health hospital, adolescents’ clinic, and private practice then follows.

## 1. Introduction

In this paper, I review some of the main art therapy models and research findings that have informed my work with children and teens over the years and organize art therapy considerations from my clinical experience. Admittedly, compared to the growing body of research practices in our field, this report is more anecdotal, and thus likely to be less generalizable to a particular mental health challenge or specific age group. However, it is my hope and belief that it articulates a working frame that is easily applicable for art therapists that are working with children and adolescents, allowing them to benefit from some our research findings and theories while particularizing the work with young clients in different settings. I specifically attempt to illustrate the usefulness of art materials, processes, and products for children and adolescents based on my clinical experiences in school settings, mental health hospitals, community clinics, and private practice.

Over the years, the use of art products and the process of art making with children have undergone extensive study as tools for assessing the normal and abnormal development of pathology in young clients. Prominent evidence-based assessments include the Formal Elements of Art Therapy Scale (FEATS), developed by Gantt and Tabone [1,2], as children respond to an art task called Person Picking an Apple off a Tree. This assessment is likely the most systemically researched and validated tool of art therapy to date. FEATS is a measurement system for applying numbers to global variables in two-dimensional art (drawing and painting). While it was originally developed for use with the single-picture assessment (“Draw a Person Picking an Apple from a Tree” (PPAT), researchers can also apply many of the 14 scales of the FEATS to other types of drawings [2].

Another very well-known model—the Expressive Therapies Continuum—developed by Vija Lusebrink, and later worked on with Lisa Hinz [3] explored the use of materials, art processes, and symbolic products. The expressive Therapies Continuum (ETC) “offers a method for conceptualizing how and why particular art interactions can be therapeutic. It provides a framework for communicating with clients, fellow art therapists, and other professionals about the therapeutic uses of art materials and processes” [3] (p. 43) and has often been used for understanding children’s and adolescents’ creative engagement focusing on the art process and materials rather on the product.

Art therapists also attempt to systemically investigate children’s use of symbols, impacted by collective and cultural aspects [4] as well as the way signs and symbols form a language that is at once unique to the individual and a manifestation of our universal focus on communication as humans [5,6].

Beyond exploring the usefulness of art making to assess and understand the needs and wants of children and teens, art therapy models have articulated the distinctive ways that art can offer therapeutic experiences and integrative opportunities for growth that is less dependent on cognitive and verbal abilities.

In her seminal book, Judith Rubin [7] articulates the main principals of working with children, typically elementary school aged children, through art therapy. She highlights the way that art materials and interventions offer children experiences of freedom as well as deep and broad expressions of self and discusses the art therapist as a figure of actual and symbolic relational relevance for the child. Rubin explains the main ways that making art, specifically visual art, integrates different levels of knowing and connects self and others (therapist, parents, peers in art therapy groups, etc.). Furthermore, Rubin emphasizes the importance of understanding specific challenges and expressive approaches and modalities while maintaining a grounded specialization in the modality one is most knowledgeable of.

While few art therapists have discussed art therapy work with pre-school children [4,8] recent work [9,10,11] has expanded the scholarship to consider the unique needs of young children, ages 2–5 years old, more systemically, suggesting a need to balance standardizing assessments, using developmental core competencies, and structuring therapy according to current evidence practice with young children and the need to offer empowering, creative, and fun opportunities for individual growth. Outside of the art therapy world, creativity researchers and brain researchers have supported the unique place of art as play and expression for the developing child [12].

Systemic considerations of art therapy work with children are plentiful and critical to understanding our interventions within the holistic realities of children. Such reflections on art therapy originally focused on family art therapy [7,13,14,15,16], as well as dyadic art therapy in form of parent-child dyadic work [17,18], couples art therapy [19], or school-based art therapy interventions [20,21,22].

Findings from family art therapy research over the years has focused on the way art production highlights participants’ roles and challenges as well as relational dynamics at the root of those challenges. Current models of parent-child dyads specifically emphasize making implicit aspects of the relationship explicit through standardized assessments [17] or connecting art therapy work with couples to evidence-based practices with clients such as emotionally focused therapy and sex therapy [19]. As work in school settings and with families is so important for child and adolescent art therapy, I will expand a bit more about each below.

Understanding art therapy work within school similarly shifted from anecdotal reports to findings from larger and more systemic exploration. McDonald and Drey [21], for example, assert that the use of art therapy in schools seems to produce some positive impacts and lack of indication of harm, and pointed to the urgent need for larger and more standardized clinical effectiveness studies of such work. When exploring the few systemic studies that were conducted in education settings, Nissimov-Nahum [22], for example, developed an evidence-based model of art therapy intervention in schools that “highlights the dual principal of conveying acceptance and directing toward change, which is applied on three levels: the child, teachers and parents, and the therapist,” and Regev et al. [23] similarly reported on the perceptions of teachers, therapists, and clients of art therapy services that were offered in schools in Israel, exploring differences between work in special educational and regular school, and advising art therapists to (1) form collaborations with staff at the educational system; (2) explain the profession and bridge the clinical and educational language; (3) stress the need for additional resources for therapy sessions, equipment, and supervision; (4) create uniform therapeutic framework; (5) ensure therapists’ adaptability and appropriate training within the schools; and (6) involve parents in the therapeutic process. Moriyah [24] also researched art therapy treatment in the Israeli system and focused on the ethical considerations of art therapy work in educational settings, similarly, underscoring the need to consider ethical dilemmas resulting from the difference between educational and more traditional clinical settings.

As noted above, family art therapy has often been used art for systemic assessments of clinical and familial needs and roles, power dynamics, and communication patterns. Treatment has focused on the need to realign these methods with family developmental stages and to look at specific clinical demands by creating space that provides psychological safety and the freedom to play, explore, and create. At times, these explorations take place with all members of the family of origin (FOO) present, while at other times, the focus of the work is on intentional sub-units of the family (parent-child art dyads, parental guidance of a child receiving art therapy, sibling dyads, or multigenerational sub-units, when clinically appropriate).

In recent years, models of utilizing parent-child dyads for assessment and treatment had become more standardized and more thoroughly researched (including [9,17,18,25], and others). Based on several large studies, Gavron and Mayseless [17] summarized findings suggesting that “the JPP enabled several dynamic processes such as pleasure and fun, bi-directionality, mutual regulation, mentalization, and mutual recognition, which together created a salient positive transformation in the relationship”.

Linesch [26] reconsiders art therapy work with adolescents and underscores engagement, empowerment, and identity as three critical clinical constructs. She illustrates these constructs through interventions that she led with marginalized adolescents, including incarcerated teens and immigrating and acculturating teens, and marginalized and gang-affiliated teens. She highlights the importance of considering cultural and systemic issues as well as the power dynamic beyond traditional developmental theories and art therapy models. Linesch, following Briggs [27,28], contextualizes the need for teens to not leave childhood, but to find a way to relate and belong to a complicated, post-modern adult reality that is embedded in feminist thinking as they consider their ethnic and racial identity in a world where oppression and privilege structure their growth. Overall, Linesch stresses the importance of considering the sociopolitical and developmental challenges art therapy practice often faces within the settings and systems in which adolescents and their families reside.

Another non-traditional setting in which children and teens are being exposed to art making and art therapy intervention includes the growing field of community art therapy. Such work sets to “promote individual and communal transformation outside of traditional health settings” [29], in such sites, the work focuses on wellness of community members through art making that supports (a) safety (structure), (b) acceptance (nonjudgment, genuineness), and (c) opportunity (authentic self, exploration, creativity, self-care). In such work, the choice of materials should take into consideration a wide range of personal, familial, and developmental needs as well as the cultural relevance of the material and the space of work and handling (storing/sharing/presenting) the art products.

As a whole, open studios and community outreach efforts postulate that creating an organic environment where community members, including children and families, can bring their authentic selves to create together increases their sense of self and wellbeing, individually and collectively. Seminal models of open studio art therapy were created by founders such as Pat Allen [30] and Timm-Bottos [31], and more recent social action frames further articulate the needs to which these models respond [32]. Community initiatives have grown in recent years as they seek to enhance the wellbeing of people living with disabilities [33], support processing grief [34] in psychiatric hospitals [35], for refugees, unhoused and displaced individuals, and survivors of domestic violence in shelters [36,37], or undertake an intensive intervention after disasters [38,39] Children and adolescents are of course part of the communities that are served by these art therapy approaches and seem to greatly benefit from the intergenerational and social opportunities that they bring. In the last couple of years, due to the COVID-19 closures and a necessary shift to Telehealth, some programs started offering online open studios that serve children as well [40].

This brief review of the literature situates this paper’s aim to categorize art therapy research findings and theoretical models into applicable clinical considerations that are based on my own clinical experience working with children and adolescents in myriad settings.

## 2. Materials and Methods: Reflecting on Learning from My Clinical Experiences

In preparing to write this paper, I reviewed my clinical files and considered my work with children and adolescents over the years. Specifically, as I looked over my clinical files, I considered my different roles as an art therapist working with children, adolescents, and families in different settings. I then looked at the theoretical models that have grounded my work and recent publications on effectiveness and relevant interventions, as discussed above. Table 1 summarizes some of the overarching considerations that might be useful for other art therapists.

To review my clinical work with children and teens, I first identified all of the primary settings in which I have worked over the years, creating categories and organizing my files into these categories. For transparency and clarity, I discuss the actual settings in which the services were delivered without disclosing information that might compromise the privacy and confidentiality of my clients.

### 2.1. School Settings

One of my formative experiences as an art therapist was my work with a unique school-based program called Share and Care, which offered art therapy services at local schools as part of the psychological trauma outreach program at Cedars-Sinai, a large medical facility in Los Angeles (https://www.cedars-sinai.org/community/programs/share-care.html (accessed on 5 August 2022)). During my years of employment with Share and Care, I was assigned to a local elementary school in the community and worked closely with the principal, teacher, and parents to explore the multiple ways individual, group, and parent-children services can support the identified needs of students. For a year, I also worked in an elementary school on the east side of LA, where families were much more impacted by immigration, challenges with legal status and employability, and violence (gang-affiliated activities) impacting children’s social lives.

In both settings, while there was no other art therapist on site, I was grateful to work alongside a devoted and open-minded staff who were able to gradually adapt schedules, goals, and procedures that were predominantly educational to ones that were more focused on the socioemotional needs that arose. A prominent challenge nevertheless was the ongoing effort to structure the work to be confidential, student-centered, and developmentally/psychologically focused in an environment where others (teachers, parents, principal) had rights and needs that were associated with information I often glean more exclusively in sessions. Another challenge was maintaining the privacy and emotional wellbeing of students who struggled with the sessions taking place in school, the firm time limit of the session, and its proximity to breaks and other classes. Clarifying priorities and goals, such as when it is best for a student to see a therapist while in school and when that decision leads to more challenges (educational, social, familial), was another focus. Finally, my work was also guided by the importance of considering various developmental uses of art and adapting to the needs of children ranging from kindergarten to fifth grade, and intentionally learning with children and their parents about ways their cultural background informed their understanding of art, therapy, and their challenges and strengths.

### 2.2. Medical and Psychiatric Art Therapy

I first learned about art therapy as a psychology undergraduate student working in a psychiatric-intensive program for teens in a large psychiatric hospital in Israel. I was employed there as a psychological mentor and facilitator. It was there that I learned firsthand the power of creative expression for teens as I struggled with not-knowing and learned the importance of setting clear boundaries while holding on to care and hope. While there, I first considered the meaning of therapeutic use of art versus art therapy. Later, in Tallahassee, Florida, I served as the art therapist in three psychiatric units in a large hospital (Tallahassee Memorial HealthCare). Unlike working in a semi-open, ongoing intensive program serving the same adolescents for months, I learned how to create brief art therapy interventions for acute situations, often not knowing if I would get to see my clients when I was there next. When I later collaborated with expressive therapists at the Children’s Hospital Los Angeles to research similarities and differences between art therapy and music therapy. During this time, I was again reminded of the importance of systemic thinking—specifically, understanding the way medical procedures and the physical environment of the hospital impacts the choice of art materials, their use, and what one does with the products. Similarly, art therapists constantly negotiated medical and psychiatric goals with humanistic and existential ones, trying to strike a balance in advocating for client’s unaccounted wants, giving voice, and providing a sense of freedom.

### 2.3. Community Clinics, Community Centers, and Open Studios

As a beginning art therapist, I constantly looked for ways to offer opportunities for the therapeutic use of art for the community in a way that supported social justice. I learned from the best—I was lucky to be mentored by Debra Linesch at the time she had created the workshop for teens at risk in the Museum of Tolerance [27] and was an active facilitator as part of that team.

I later facilitated art therapy work with pregnant and parenting teens for a couple of years through the Helen B. Landgarten Art Therapy Clinic, which served adolescent girls between the ages of 13 and15 who attended Thomas Riley High School, an alternative school in South Los Angeles. I also had the unique opportunity to be part of the art therapists of Katrina Through the Eyes of the Children (https://www.nytimes.com/2007/09/17/arts/design/17ther.html (accessed on 5 August 2022)), offering an intensive art therapy workshop for two to three days bi-monthly for a community of Hurricane Katrina survivors who had been evacuated from New Orleans to a temporary housing (Renaissance Village) in Baton Rouge. In recent years, I co-directed community outreach projects, both physical and through Zoom, offering art therapy services in Latin America and Israel, which similarly continue to inform my thinking.

Specifically, seeing children and adolescents in community and non-clinical settings, where the focus of the work is often layered and includes shared socio-environmental strife impacting a community is humbling and life-affirming for me. Witnessing the power of art to hold, explore, and communicate how each of us is so different and yet belong so deeply to our community, and how our context shapes us, is at the core of my belief in our work. Working in these setting also continuously sheds light on my own privileges and cultural assumptions and sustains the imperative to be flexible yet consistent and professional within fluid, constantly changing settings with limited resources.

### 2.4. Family/Dyadic Art Therapy

As an art therapist in private practice for the last decade, I have had the opportunity to serve families and parent-child dyads. The focus of art psychotherapy with families and parent-child dyads is, naturally, the relationship AND the individuals rather than the individuals primarily, as is often the case in other forms of therapy. Art making within these settings has served me as a therapist in pacing (myself, my clients), holding all experiences as valuable as they are captured through the art and can be considered later in therapy.

As I reviewed notes from different families and dyads I had worked with, the importance of creating a shared understanding of the purpose of therapy emerged. Art had often showcased the strengths of family members and the dynamic (and challenges that were associated with that dynamic) among them in a way that was concrete and integrative, personal, and shared.

Offering services in private practice also asks for different involvement of the therapist as a business owner as well as the sole holder of legal and ethical responsibility. These aspects of private practice as a setting often limit, shape, or stretch the roles of the therapist with the adults who pay for these services and can impact their response to art and verbal interactions.

### 2.5. Individual (Child) Art Psychotherapy

Again, when reexamining my work with children and teens in my private practice as individuals, the negotiation of communication and control of parents as the consenting adults comes up. With some parents, the challenge was to engage them more in therapy and encourage more of an understanding of their child or teen’s needs, while for others my role was to assist the child or teen in creating a discrete and autonomous space. In this setting, I often anchored my interventions in relational and developmental theories, attempting to assert my position as a trusted adult friend for the child or teen even as I held on to the importance of the parents in their child’s wellbeing.

Within this context, art—exploring materials, allowing young artists to experiment and practice regulation, working through frustration, illuminating inner experiences—was always my most helpful tool. Storing artworks overtime often helped communicate to both the child and the parents who were not present in all sessions what journey their children were on.

### 2.6. Group Art Therapy

I led groups in many of the above settings (schools, hospitals, community outreach workshops); yet I think it is worth considering the unique aspects of creating a clinical group with the focus of the group as the healing environment rather than as a byproduct of a system in which many clients need to be seen. In other words, I explored children’s and teen groups whose setting was essential to promoting the goals. For example, when offering groups for teens that were housed in a residential facility for adolescents that were at risk (Penny Lane, Los Angeles), the group of participants also lived together and were essentially a sub-family of a sort. There, increasing empathy and communication supported problem solving of daily challenges.

Similarly, for children that were temporarily housed together in an inpatient psychiatric hospital, art therapy for teen mothers, or for adolescents that were placed in juvenile detention offered a normalization and validation to their journey. So, understanding the merit and limitations of each group directed the goals as well as the art interventions that were used.

### 2.7. Art Therapy Assessment

Although art therapy assessment can take place in any setting in which children and adolescents are seen by an art therapist, as I reviewed my clinical work, attempts to use more formalized assessments stood out from the process of using art making for ongoing therapy.

Specifically, I recalled how I attempted to study art therapy assessments for 0–5-year-olds while integrating evidence-based practices, play therapy models, and developmental frames [41]. I also remembered the use of formal assessments such as the FEATS [2] and Kinetic Family Drawing [42] when trying to learn from teens in schools and in Central Juvenile Hall (Los Angeles) about their perspectives, and the use of Helen Landgarten’s Family Art Assessment [14] when working with new families. These assessments provided structure both for myself and for my clients during our first meeting, set the tone of professional yet creative engagement, and yielded rich information about main interests, challenges, and responses to art upon which to build.

## 3. Results/Case Illustrations

As I organized my insights, I noticed two overarching considerations that continuously inform my thinking: First, considerations of clinical settings (where the child is seen, referred, when and where therapy takes place, considering the bio-psycho-socio-cultural affiliation, etc.) in which children or adolescents are seen. And second, considering how children and teens utilize art materials, art processes (interventions), and art products within the developmental and environmental conditions they experience. Accordingly, in the table below, I attempt to summarize some of the main points that emerged from the research and theoretical frames that were discussed above that were meaningful in my clinical experience. Later, I offer brief case vignettes as examples of applying some of the noted considerations within the different settings. These derive from my experience as an art therapist with the hope they can be considered an illustration as well as support for other art therapists’ application of their own work.

In the section below, I illustrate the way different settings and systemic considerations are entwined with reflections for using art materials and art interventions (process) and the resulting art products in art therapy with children and adolescents. In other words, while the first section presented research findings and theoretical models for art therapy with children and adolescents, then summarized the clinical considerations, those considerations are now applied to concrete examples and case vignettes in this section.

### 3.1. Working in School Settings

As explored above, evidence-based practice of art therapy in school settings is now well anchored in substantive research [20,22,23] and often highlights the unique place that art therapy creates within a complicated system involving children, teachers, parents, and administrators. Both systemic and developmental variables impact goal-setting that is often relevant for both educational/academic needs as well as mental health ones. The choice of art materials, time, and space in which sessions take place, as well as the available art products are often an outcome of negotiation between the therapist and other school players. The role of the therapist in a unique relationship and as an adult trusted friend [26] must also be negotiated with the fact that the therapist must communicate with the child’s parents, their teachers, and report formally as part of the school system.

An illustration of the above considerations within the school system is demonstrated in my work with D, a 10-year-old child who was originally referred to art therapy by the school principal due to repeated bullying and violent behaviors and failure to thrive academically. As a school-based art therapist at the time, I had an initial assessment with him and a follow up conversation with his mother, a single parent, in which many insecurities and hesitations about the school and how he could succeed in it were raised. It was clear to me that a main goal of therapy was to increase D’s sense of safety and trust in the school system and in his interaction with me, and that he desperately needed to be seen as capable and valued.

**Figure 1:**
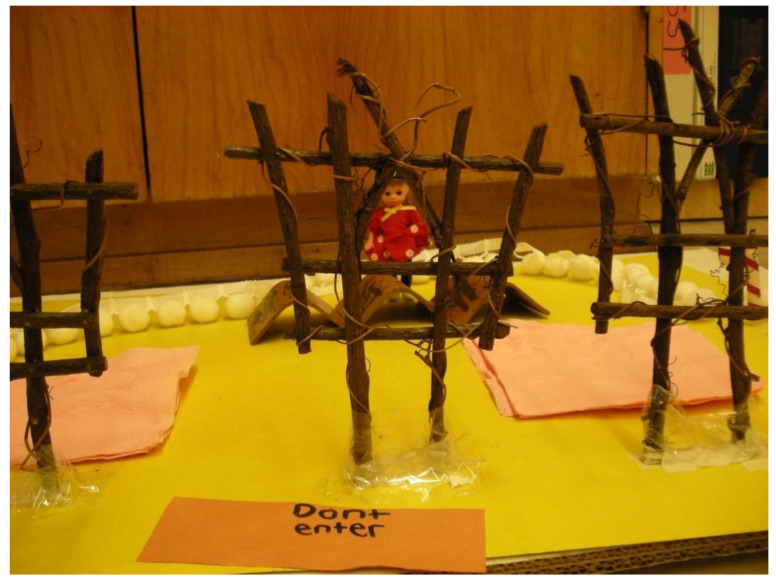
Working with a child in the school system.

The art piece (Figure 1) shared here allowed D to use materials that were novel and appropriately challenging for his age (hot glue gun) and produced an art product (construction of found objects) that he was proud of (he did not feel comfortable drawing). The fact D could choose the materials enabled me to support the creation of a scene he developed and controlled, which fostered his sense of safety and freedom to explore and allowed him to reveal aspects of himself—in essence, his fragility—that he rarely presented in the classroom. The resulting three-dimensional piece allowed us to interact with the child behind the “Don’t enter” sign while keeping him safe psychologically behind the play and metaphorical figure. His ability to share more vulnerable sides helped me better advocate for his needs in the classroom and generate more empathy from his school staff.

### 3.2. Medical and Psychiatric Art Therapy with Children

Some of the considerations in working with children and teens who are hospitalized (for either medical or psychiatric reasons) bear systemic similarities to working in educational settings; for example, the system of care (program, hospital), the need to consider safety and limited resources in material choice, as well as the storage of art materials and art products. It is also important to think about team approaches (working with a treatment team) and holding a private safe space for a minor, keeping in mind that a therapist is bound legally, ethically, and clinically to have some transparent communication of progress with caretakers.

Clinical goals often also need to be developmentally attenuated, but the focus of the work tends to be on very brief crisis intervention. In hospital settings, art often provides opportunities to play and summon joy and distraction from physical and psychological strife, even as art contains the emotional gravity of the situation and helps the child face the challenges that brought them to be hospitalized. Notably, art therapy in hospitals is often structured as a one-time intervention with no clear plan for the child to be seen again by the therapist.

**Figure 2:**
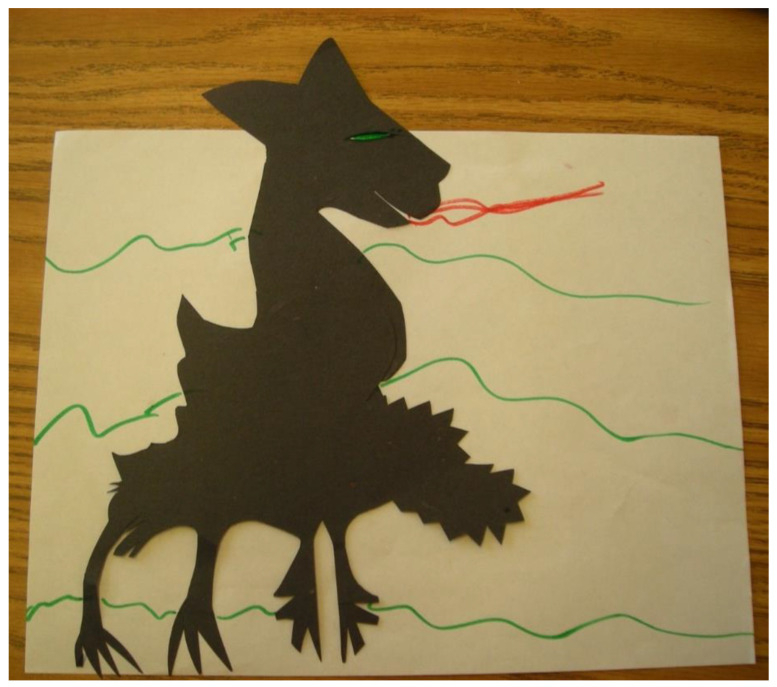
L’s cut paper monster emerges before her surgery.

Preliminary studies comparing the effectiveness of art therapy and other expressive modalities, such as music therapy, suggest that the child’s source of agony—for example, struggling with physical pain compared to alleviated anxiety and depression—might be better treated by one modality versus another [43]. In Figure 2, for example, a 15-year-old child (L) who was meeting the art therapist before a scheduled surgery in her pelvic area was cutting shapes from black cardboard paper, which she said was a way to play and relax. One of the shapes that emerged developed into a monster which she later pasted onto a white paper as a background. As she continued to embellish the overall creation, a shift occurred from tactile a focus to a more emotional cognitive, and later symbolic, processing [3], and she was more able to process the image to communicate her fear of the illness, the surgery, and their potentially dangerous outcomes.

### 3.3. Working with Children and Teens in Community Centers and Open Studios

More and more, art therapy work takes place in non-traditional settings that benefit communities and individuals through less clinical frames such as open studio and community outreach models [29,35]. Work of this sort often takes place in community centers, NGOs, shelters, hospitals, community events, and places of worship and gathering. As such, the settings provide a more natural and less confidential, less private meeting space, and the art making becomes a way to enhance wellness and express oneself while engaging within a relevant community. Some considerations, therefore, include having a clear rationale and clinical goal with an identified community where one’s shared experience can be meaningfully expressed. Considering the space that is available and how to open the doors to the public in a way that at once invites all who can benefit yet maintains structure, safety, and intimacy is an important challenge. Working with prominent community leaders to make sure the setting and interventions are culturally appropriate, and continuity of care is available within the community should additional services be required are also of great importance.

I had been fortunate, personally, to offer art therapy services of this nature as part of several wonderful community outreach projects, including Karla Leopold’s initiative “Katrina Through the eyes of the Children”, community art therapy workshops that were offered in Mexico at the Instituto Mexican de la Mujer” in San Miguel de Allende, Mexico, and—in the last few years—offering art therapy workshops virtually in Latin America through an art therapy international collaboration of three art therapy programs from the United States, Mexico, and Israel.

**Figure 3:**
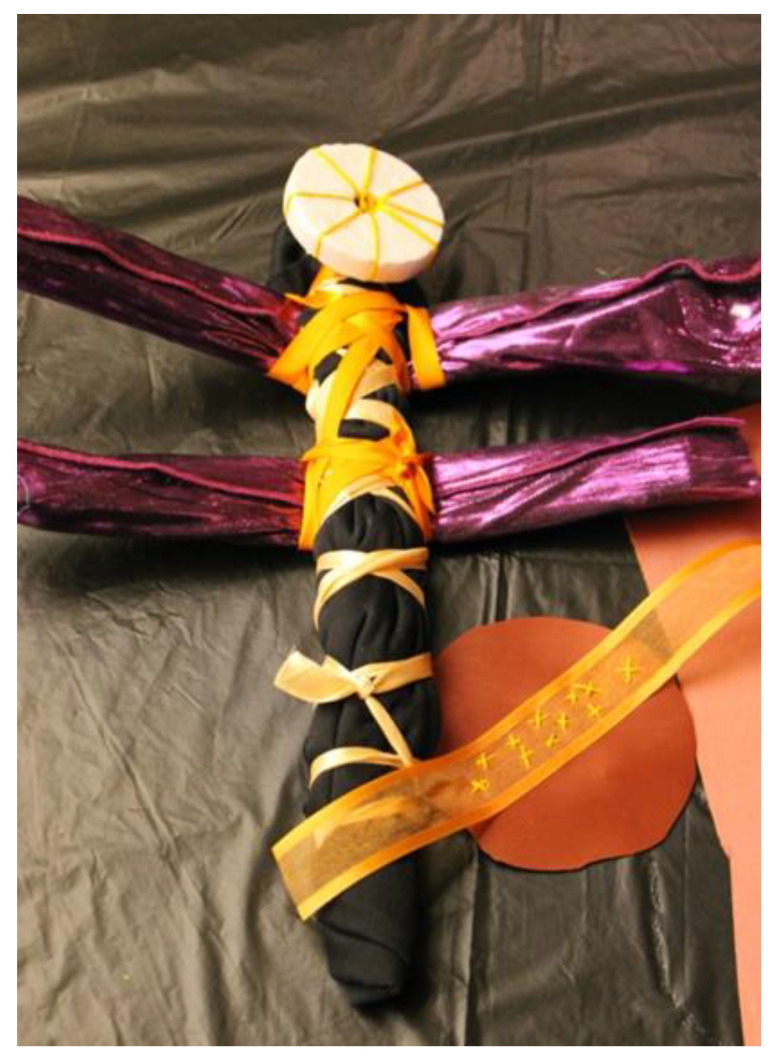
R and F create a dragonfly in an open studio in a shelter/community work.

In my experience, working in non-clinical settings requires the therapist to remain professional yet very flexible and open. The focus on relationship forming (with community members, and theirs’ with art making and art products) are supported by safety, acceptance, and opportunity [29], which are held and modeled by the therapist. When R and F, a mother and daughter who had been displaced due to Hurricane Katrina, attended an outreach open studio I facilitated, they first arrived in my space searching for water and a place to sit and eat. Here, already, is an example of the clinical settings being stretched—they joined an already working group of families and individuals engaging with available materials and minimal direction. They were welcomed to join us and eat, and of course to explore the materials and join the art making. I introduced myself and the team and explained that our purpose in art making was to relieve stress and connect with one another after their terrible experiences of displacement. I then described our terms of engagement with materials and each other in the room and after they observed quietly and ate, they created a dragonfly (Figure 3) from sticks, fabric, and so on. As they worked, I could see that their interaction with the materials and each other lifted their spirits. They discussed color choices and reminisced about a dragonfly they used to see. Slowly but surely, they began to engage with other participants, sharing stories of their homes, of the trauma brought by their displacement, and ultimately came back to make more dragonflies several days in a row. The dragonfly became a symbol they could carry with them, a symbol of hope and transition, which they repeated and perfected, while the setting offered a place of freedom, mastery, and connection in a time of great uncertainty and strife.

Working collectively, in my experience, offers an additional therapeutic value through a sense of belonging and having connections and support from others who become consistent witnesses to their joys and struggles. As noted earlier, art therapy that takes place in a non-clinical community space (whether temporary housing, a school, a community center, a gallery, etc.) often has positive impacts on the individual and the community when children work together with others on collective projects (such as a mural, a shared exhibit, etc.). In such community projects, the children’s voices and needs become amplified by art making and the images bring pride and empowerment beyond the sterile and confidential setting of traditional therapy.

### 3.4. Working with Children and Their Families

There is so much that had already been written about working with families in art therapy, parent-child dyads, and so on. My intent here is thus to highlight the considerations that stand out to me—from all the models and research I have explored—as most relevant to my own work with families. Beyond general considerations of materials (so they are appealing to the adults as well as he children or teens) is the need to pay attention to relational dynamics. Art making, both the process and product, showcases initiation and hesitation, decisions about engagement, collaboration, imitation, and boundary forming. The art also elucidates cultural and systemic considerations of needs and values underlying clients’ perceptions of health, therapy, individual identity, and family or community belonging, all of which can be shared between family members or illuminate gaps between family members while the art supports processing these dynamics.

**Figure 4:**
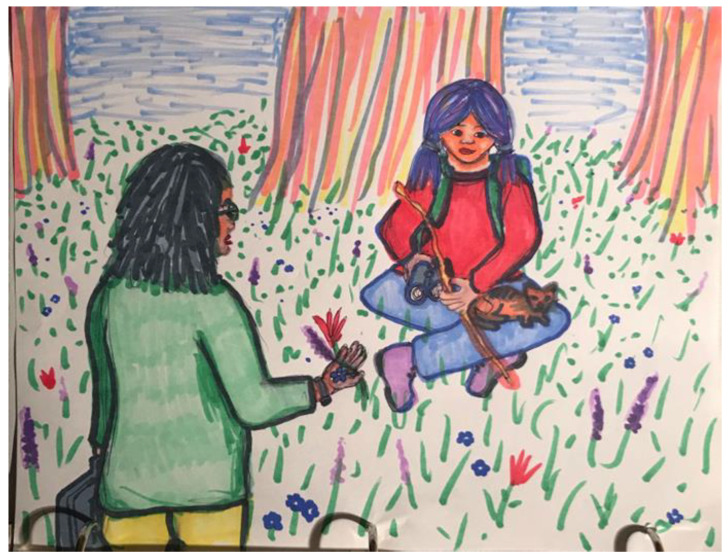
A mother-daughter dyad explore differing gender identities through art.

An example of such a use of art can be seen in Figure 4, an image a mother created for her daughter in a joint session in which she addressed her hopes and fears that were related to her daughter being a 12-year-old girl. Much of the conflict between them revolved around the mother’s behavioral expectation from her daughter, which diverged greatly from what the daughter wanted and what she perceived as expected and validated by her peers. When exploring her own image and reflecting on it, the mother could see that in her drawing her daughter seemed younger than her current age (realizing part of the challenge was developmental). She could also see that her own wishes and experiences as a girl and the difficulties of becoming a woman were projected on her daughter. When both mother and daughter could process these insights, a greater empathy emerged, and the image itself was later replicated and modified—a reflection of their evolving perception of themselves and their relationship.

### 3.5. Working with an Individual Child in Private Practice

I love working with children and teens through art. There is magic and play and an intimacy of deep knowing that is hard to explain. I sense no verbal therapist, as sensitive and wise as she is, gets to experience the internal life of a child or a teen in the same way that is facilitated by playing and creating as the child leads. When we utilize the art therapy setting with opening and closing rituals, consistency of engagement and materials, and a clear structure of time and place, children and teens thrive in the freedom and empowerment of validated self-expression.

**Figure 5:**
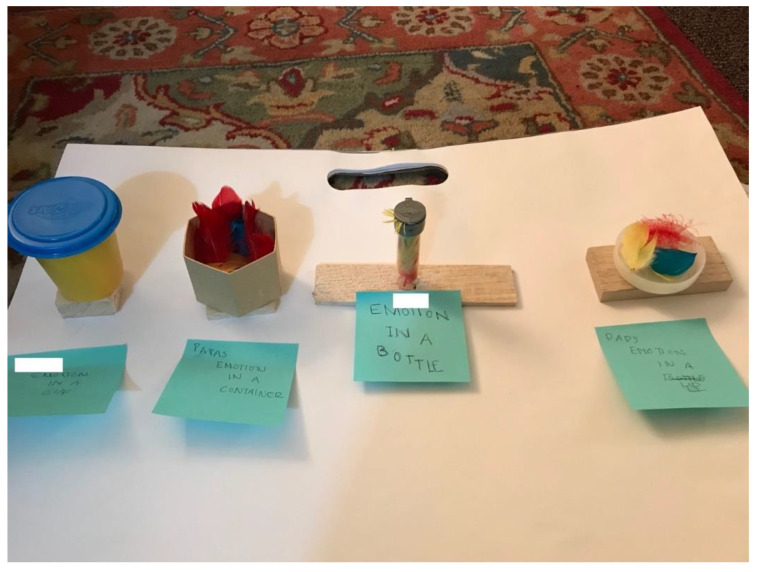
A’s emotions in a bottle (private practice individual art therapy).

Children and teens can get stuck in dark corners of shame and guilt, get stuck facing families or systems that do not see them or do not work for them. The art, and the art therapist, can shed light into the deserted and unexplored spaces within. Figure 5 depicts A’s bottled emotions. He presents each member in his family through a different container and the way they contain or release their emotions. A came to see me after a very challenging family crisis that had a public presence and led to the separation of his parents. He could barely say anything at the beginning and was always careful to be positive and diplomatic in all of his responses. Art and made-up characters and metaphors allowed some distance from the realities of his life, giving him a bit more freedom of expression. Only after a few sessions was he able to create the bottled emotions piece (Figure 5), carefully letting me know how pent up and sealed within himself he feels.

### 3.6. Working with Groups of Kids or Teens

Children’s and teens’ developmental needs are increasingly centered around peer interactions, as they continuously explore and modify who they are relative to others and as part of peer and family formations. As noted in the literature, art therapy groups for children and teens that are suffering from challenging life experiences (grief and loss, divorce, illness) are common, as are groups that are centered around mental health struggles (attention disorders, behavioral challenges, addiction disorders, or anxiety). For adolescents, because of the developmental stage of individuation and increasing sociopolitical awareness, the reflective and expressive aspects of art making serve as tools for engagement, empowerment, and identity formation [26]. All the above were central to my own experiences as a therapist working with teens in residential settings, in psychiatric hospitals, and with parenting and pregnant teens. As a therapist working with adolescents, the art helps me with the intensity and multiplicity of teens’ emotional experiences even as I insist on the boundaries in their relationships with me and with other group members, so I can consistently and compassionately support their exploration of self and others. The art making and exploration create the frame of the group within which group members have the freedom to engage, explore, express, resist, negate, and push back [41].

**Figure 6:**
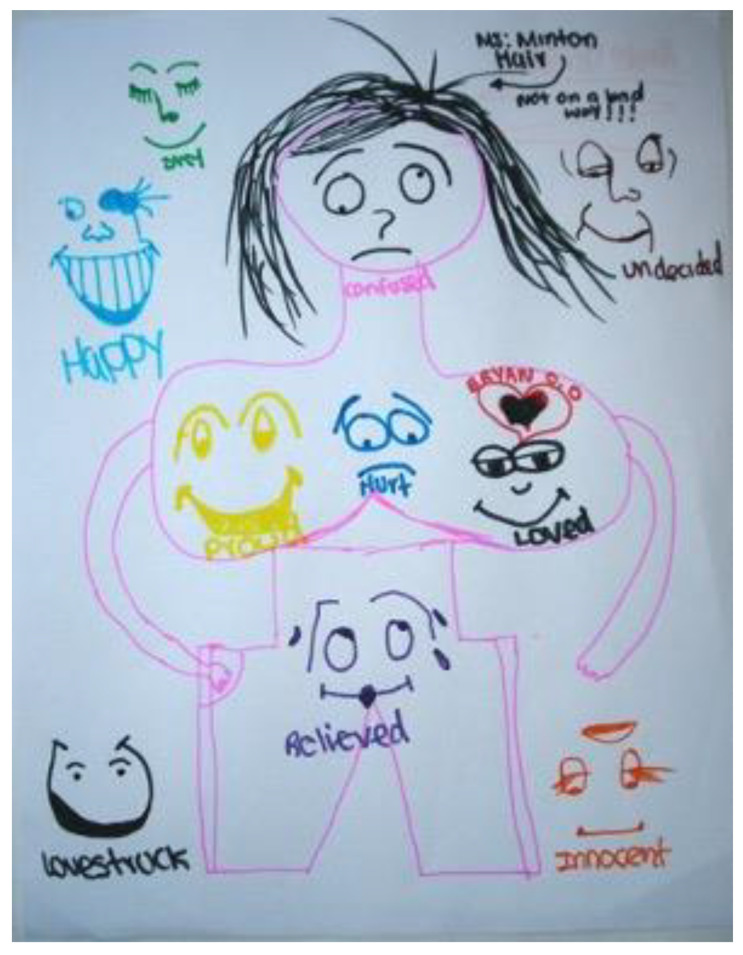
Normalizing and validating with peers: pregnant and parenting teens’ groups.

Figure 6 demonstrates the work of a 16-year-old who presented to our parenting and pregnant teen groups with a hostile attitude and a clear need to test the boundaries that were set by engagements with peers. Redirections, attempts to give her a (verbal) voice and place in the group did not make much difference. However, after sitting down with a list of emotions (provided) and an invitation to express the emotions that were stored within her body, a powerful image emerged. As she sat with the multiplicity of experiences within her and recognized many of those in her peers’ images as well, she found meaning. In the group, in the art, and in the presence of her peers.

### 3.7. Utilizing Art Therapy Assessment in Art Therapy and Family Therapy

**Figure 7:**
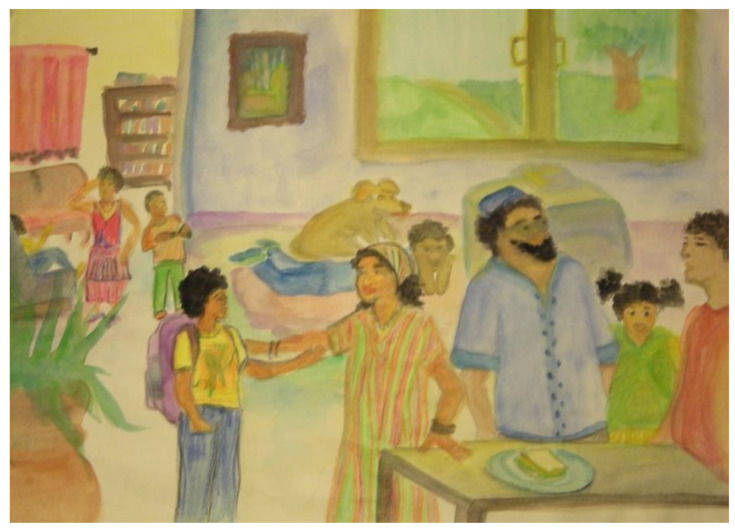
Kinetic Family Drawings with teens—cultural considerations of FOO.

When working with clients of any age, it seems to me that we therapists must constantly consider the known and unknown, the general and the particular, the universal and personal. Thus, when a client suffers from a mental health or a pathology, we must know something about the norm (behavior, mood, experience) from which there is a diversion, typical manifestations, etiology, and prognosis, and hold onto those as we attempt to understand the person in front us (and their behaviors, experiences, and symptoms). When we work with children, the need to constantly assess and evaluate the needs of our clients is doubled by the need to consider their developmental stages as typical and atypical. Naturally, any assessment of merit must consider the client’s educational environment, socio-cultural background, family and living situation, and so forth,

Although I am not trained as a psychologist or an administrator of most psychological tests, I do find that using our standard art therapy assessments can be immensely helpful in holding the unique and particular aspects of clients’ experiences while also paying attention to normed features or socio-cultural aspects. A simple example is my use of the kinetic family drawing (KFD) assessment with a 15-year-old teen of Middle Eastern background (Figure 7). The simple guidance—“Draw you and your family doing something together”—as part of our initial time together opened the door to exploring his personal memories of his childhood home and his story of immigration. It also offered a glimpse into the shared cultural understandings of family of origin (FOO), which was defined differently than in the United States, an exploration of a typical living environment for him, and the way touch and space representations differed from the typical KFDs I see.

## 4. Conclusions

This paper attempted to integrate clinical considerations through a systemic exploration of my clinical work as an art therapist with children and adolescents over the years, informed by current research and the main art therapy models that have shaped my thinking. As I reflected on this work, three main points that have guided my art therapy work with children and adolescents emerged:

(1) Art offers a uniquely profound tool for children and adolescents, one that is easily adaptable to different developmental stages. Choice of materials, ways of facilitating the process of art making, and utilizing the art products to augment the therapeutic experience also stood out.
(2) Systemic thinking enhances the impact of therapy for minors—one has to particularize with whom and how we work based on the therapy setting, and the bio-psycho-socio-cultural aspects of client’s experiences, as I attempted to illustrate above.
(3) Art therapists hold therapeutic structure that fosters freedom for children to reinvent themselves through creative engagement. Put differently, an art therapist offers art opportunities while being an adult trusted friend, as conceptualized by Brems and Rasmussen [44]. They often must hold a clear structure of engagement that keeps the child or teen safe, physically, and psychologically. At the same time, she offers empathy and validation, curiosity, and flexibility steeped in commitment to the freedom creative expression summons from each child or teen.

The three points above are general ones, and none of them is novel in the field of art therapy. Nevertheless, my hope was that a review of the breadth of art therapy work with children in conjunction with clinical considerations and illustrations of such work in a wide range of settings and developmental stages would be helpful. Mostly, as a therapist who truly appreciates what art does for children therapeutically, my take-home reminder is that art is fun just as much as it is seriously helpful for kids and those who care for them.

## Figures and Tables

**Table 1:** Working with Children in Context: Summarizing art therapy research findings and theoretical models into applicable clinical considerations.

Setting	Art Materials	Art Process	Art Products
School settings	- Materials that are accessible and safe in educational settings - Offering new techniques and materials that are not often available in classrooms and empower the child (such as supervised work with hot glue gun)	- Providing opportunities to engage in a process that offers safety and freedom - Considering the child/teen’s ecological and systemic needs and engaging parents, teachers, and peers to enhance therapeutic impact	- Focus on art products as “bookmarks” or “steppingstones” reflecting diversity of internal and external experiences to be integrated (radical acceptance) - Model caretaking of art products as extension of self and relationship
Medical and psychiatric art therapy	- Materials that are accessible and safe for hospital - Art materials that increase expressive opportunities but do not cause further stress or sense of physical or mental limitations	- Providing opportunities for non-medical play, joy, connecting to hope, health, and processing impact of fear, sadness, and trauma within a wellness frame - Understanding transitional nature of art therapy sessions as a one-session brief therapeutic experience	- Understanding the transitional nature of art therapy in a transitory space: 1. The possibility for the product to transform the environment through small creations, and 2. Practicing non-attachment and acceptance of the often-limited ability to store and transport physically.
Community clinics, community centers, and open studios	- Considering individuals’ goals within their community and its resources impacting choice of materials - Exploring ways community and public engagement and interaction can enhance therapeutic goals (finding spaces for exhibitions, storage, and clinical practice that empower community members and respond to needs)	- Exploring ways community and public engagement and interaction can enhance therapeutic goals (through exhibitions, collaborative/intergenerational work, team-work, etc.) - Utilizing therapeutic process and structure to build on the community strength and offer culturally appropriate interventions	- Considering the meaning of artifacts created in art therapy sessions as potentially meaningful to the community and consider the possibilities of shared growth through creative collaborations - Considering the meaning of privacy/confidentiality, as well as use of care (extended families?) and the role of the therapist and their relationship to community leaders to enhance sustainability
Family/Dyadic Art Therapy	- Considering materials that are appropriate and engaging for age range of participants. - Creating work environments and spaces for collaborations as well as parallel work	- Paying attention to relational dynamic: initiation and hesitations, decisions about engagement, collaboration, imitation, and boundary forming - Exploring similarities and differences between verbal and non-verbal aspects of engagement	- Using reflective and verbal tools to process and validate individual and shared experience - Support exploration of art products to increase self and other’s understanding, connect here-and-now to ongoing relational experiences and therapeutic goals
Individual art therapy in Private Practice	- Offer choice of materials that does not overwhelm client yet allows for expression of needs and wants, allows for experimentation and hesitation as well as familiarity - Create rituals of presenting materials and storing them away that creates safety and structure, while allowing expressive freedom and pacing during the session	- Developmental/age considerations to instruct use of creative intervention, place of verbal/cognitive processing, and therapeutic goals - Cultural and systemic considerations of needs and values underlying client’s perception of health, therapy, individual identity, and family/community belonging	- Exploring meaning of art products for individual clients within the confines of confidentiality and standards of care for minors - (e.g., consider how and when to encourage sharing art products with parents, how to empower child/teen to own communication, and what to do with art products as therapy comes to a close)
Group Art Therapy	- Materials and space considerations that engage a group of children or teens in an age-appropriate manner, and that offers freedom to explore and express while maintaining structure and safety	- Creating sound structure (through opening and closing rituals, consistency of rules of engagement, and responding clearly to attempts to breach or assault set structure) while offering freedom to create and express with unconditional support and validation	- Considering movement between shared group and individual presentation and discussion of artworks to sustain and reflect group members’ needs of belonging as well as separation-individuation within a group
Art Therapy Assessments	Consider appropriateness of standardized or un-standardized Use of Materials Art Therapy Assessments (i.e., PPAT, KFD, JPC)	Considering clients foci/level of work and possibility of integration (ETC, self and other, cultural aspects)	Standardized or un-standardized understanding of products within developmental and cultural/ecological realities

## Data Availability

Data available on request due to restrictions of privacy or ethical stipulations. Namely, the data presented in this study are available on request from the corresponding author. The data regarding specific cases and contexts of treatments are not publicly available due to ethical and clinical obligations of art therapy practice.
